# Hypertension as an Underlying Cause of Headache or Dizziness and the Usefulness of Choto-San for Both Diagnostic and Therapeutic Purposes

**DOI:** 10.7759/cureus.66212

**Published:** 2024-08-05

**Authors:** Ryuzaburo Kanazawa, Noboru Kuniyoshi

**Affiliations:** 1 Neurosurgery, Nagareyama Central Hospital, Chiba, JPN; 2 General Internal Medicine, Nagareyama Central Hospital, Chiba, JPN

**Keywords:** dizziness, headache, potential hypertension, choto-san, kampo

## Abstract

Background

Though headache, head discomfort, and dizziness are major complaints in neurosurgical outpatient departments in Japan, these nonspecific complaints are sometimes troublesome to treat, and most symptomatic treatments are not always sufficient to resolve patients’ complaints.

Objective

This retrospective study was conducted to identify potential hypertension underlying symptoms relating to the head by prescribing choto-san, because patients with such symptoms were found to have relatively high blood pressure, and we realized that Kampo medicine was potentially effective for resolving the patients’ conditions.

Methods

A total of 171 patients making their first visit to the neurosurgical outpatient division of our institution from January 2020 to June 2022 were investigated retrospectively. Symptoms were classified into three categories: headache, head discomfort, and dizziness. The effectiveness of choto-san, the rate of potential hypertension, and whether improvement in symptoms had a strong relationship with the prescription of choto-san were investigated.

Results

Choto-san significantly improved outcomes, with an odds ratio of 3.13 (95% confidence interval 1.83-5.35, p<0.001) for choto-san and 5.50 (95% confidence interval 1.24-24.4, p=0.025) for antihypertensives. The rate of choto-san prescription was significantly higher in patients who were diagnosed with hypertension (test of independence p<0.001). Choto-san was thought to be the most effective for the aforesaid symptoms with accompanying potential hypertension.

Conclusion

Hypertension was shown to be one of the main causes of various nonspecific complaints. Choto-san was an effective medicine not only for improving patients’ subjective symptoms but also for identifying potential hypertension, which may lead to the prevention of cerebrovascular diseases.

## Introduction

Headache is one of the most common symptoms encountered in the neurosurgical outpatient clinic. We frequently see patients with nonspecific complaints, such as neck stiffness, dizziness, head discomfort, and tinnitus, and they are occasionally troublesome to treat. Some painkillers are not always effective for these patients, so it is important to identify the true cause of these symptoms. We have often observed that outpatients presenting with these symptoms also have concomitant hypertension. Therefore, this retrospective study was conducted to investigate whether hypertension was an underlying cause behind these medical symptoms and whether choto-san (a Kampo medicine) was effective for these symptoms and the detection of hypertension. Choto-san may be useful for such detection because it is thought to manifest its effect by lowering blood pressure, resulting in remission of various symptoms derived from potential hypertension.

## Materials and methods

A total of 171 patients who made their first visit to our neurosurgical outpatient division from January 2020 to June 2022 and complained of headache, dizziness, neck stiffness, nausea, head discomfort, unsteadiness, tinnitus, etc. were investigated retrospectively. The background of patients was followed in relation to the presence of anti-hypertensive prescription, hypertension, and stroke as familial history. All medical information was collected from medical records by a staff member (S. T.) of our medical cooperation division, who was not informed of these patients’ personal information. The information about the patient's age, sex, chief complaint, whether or not the patient was taking antihypertensive medication, family history of hypertension, etc. was extracted by the staff from the medical records who classified the symptoms recorded in the medical records according to the classification shown in Table 1.

**Table 1:** Classification of patients’ complaints

Category	
Headache	All symptoms including “pain” inside patients’ chief complaint
Head discomfort	Foggy, not clear, neck stiffness
Dizziness	Unsteady, tinnitus, unstable

Classification of symptoms

Symptoms were classified into three categories: headache, head discomfort, and dizziness. The classification is summarized in Table 1. The average age was calculated for patients with each symptom and patients with two or more complaints. Choto-san was prescribed at a dose of 2.5 g, twice a day (before breakfast and dinner time, respectively). The recommended dosage is 7.5 g/day, but as patients often forget to take the medication three times a day, the dosage was changed to twice a day in the hope of ensuring continued medication.

A good outcome was defined as confirmation that there was an improvement in the chief complaint or as the continuation of the prescription because of its effectiveness based on the medical record. This was also judged by the staff rather than by the authors, based on the medical records and the definitions mentioned above. Triptan preparations and valproic acid were classified as medicines for migraine, and other general painkillers (loxoprofen, acetaminophen, etc.) were classified as headache medicines. Hypertension was defined based on the home blood pressure as follows: systolic blood pressure (SBP) > 140 mmHg and/or diastolic blood pressure >90 mmHg. To exclude white coat hypertension, home blood pressure was measured for approximately two weeks after the first consultation, and the diagnosis of hypertension was made based on these values. The apparent existence of hypertension at the patients’ first visit could approve the prescription of choto-san as a diagnostic treatment. Regarding the diagnosis of hypertension, the staff made the judgment based on the blood pressure values recorded in the medical records.

Classification of prescriptions

The prescriptions were classified as choto-san, antihypertensives, migraine medicines, headache medicines, and others, and the rate of each prescription was investigated. The final prescription given to each patient was used because many patients had multiple prescriptions.

Statistical analyses

The relationship between a diagnosis of hypertension and the number of choto-san prescriptions was analyzed with a test of independence. The contribution of choto-san to symptom improvement was analyzed with a test of independence. The relationship of each prescription to each complaint was analyzed by principal component analysis. The relationships of painkillers, migraine medicine, antihypertensives, choto-san, and other medications with good outcomes were analyzed by logistic regression analysis. Logistic regression analysis was also used to identify the most significant factor among headache, head discomfort, dizziness, two or more symptoms simultaneously, migraine, and diagnosis of hypertension having a causal relationship with choto-san.

A value of p<0.05 was considered significant. Bell Curve for Excel version 4.02 (Social Survey Research Information Co., Ltd. (SSRI), Tokyo, Japan) was used for the analyses.

## Results

A total of 171 patients who came to our division as first-time patients from January 2020 to June 2022 and whose complaint was a headache, feeling dizzy, shoulder stiffness, nausea, head discomfort, lightheadedness, etc. were investigated retrospectively. Fifty-seven were male (median age 57.7 years) and 114 were female (median age 61.4 years). The prescription of hypertensives in advance was observed in 42 patients (24.6%). The familial history of hypertension was also confirmed in 42 patients (24.6%). Stroke as familial history was shown in 32 patients (18.7%). Most patients did not think that the cause of the chief complaint was derived from hypertension.

The chief complaint was headache in 107 patients (62.6%), head discomfort in 34 (19.9%), and dizziness in 51 (29.8%). The median age was 58.0 years for headache, 54.7 years for head discomfort, 69.2 years for dizziness, and 56.3 years for two or more symptoms. The median age was significantly higher for dizziness (p = 0.0172, ANOVA) (Table 2).

**Table 2:** Frequency of each symptom The average age of patients with dizziness is higher than those with symptoms in other categories (ANOVA). Discomfort: head discomfort, two or more: multiple symptoms present simultaneously. P<0.05 is denoted by an asterisk, and p<0.01 is denoted by a double asterisk.

	Headache	Discomfort	Dizziness	Two or more	p-value
N (171)	107 (62.6%)	34 (19.9%)	51 (29.8%)	37 (21.6%)	
Age, years (median)	58.0	54.7	69.2	56.3	p = 0.0172*

The following were prescribed: choto-san was prescribed to 86 patients (50.3%), antihypertensives to 22 patients (12.9%), migraine medicines to 19 patients (11.1%), headache medicines to 46 patients (26.9%), and other medications to 23 patients (13.5%). The rate of choto-san prescription was significantly higher in patients who were diagnosed as having hypertension (test of independence, p<0.001).

The number of improved outcomes was significantly higher in patients who were prescribed choto-san than in patients given other prescriptions; therefore, the treatment efficacy of choto-san was thought to be relatively higher than that of the others (test of independence, p = 0.0163).

**Figure 1:**
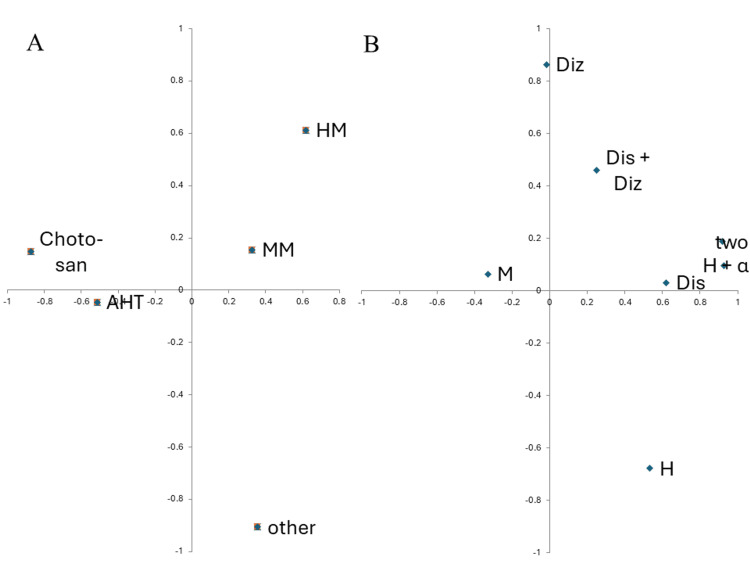
Relationship between each medication (A: left) and each symptom (B: right) Antihypertensive and pain relief characteristics of choto-san (A). Migraine separated from the three main symptoms (B). HM: headache medicine; MM: migraine medicine; AHT: antihypertensive; Diz: dizziness; Dis: head discomfort; M: migraine; two: more than one symptom of headache, head discomfort, and dizziness. H+α: headache plus head discomfort or dizziness; H: headache

Choto-san and antihypertensives were closely related, and they had weak relationships with migraine medicines, headache medicines, and others on principal component analysis. The aforementioned three symptoms (head discomfort, dizziness, and headache) were completely different from the symptoms of migraine (principal component analysis) (Figure 1).

Choto-san and antihypertensives contributed to outcome improvement. The odds ratio for choto-san was 3.13 (95% confidence interval 1.83-5.35, p<0.001), and it was 5.50 (95% confidence interval 1.24-24.4, p=0.025) for antihypertensives (Table 3). The symptoms that were improved after treatment were headache, dizziness, and symptoms of migraine. The odds ratios were 1.96 (95% confidence interval 1.20-3.19, p=0.007) for headache, 1.95 (95% confidence interval 1.02-3.7, p=0.042) for dizziness, and 4.73 (95% confidence interval 1.03-21.7, p=0.0455) for migraine (Table 4). The most common reason for prescriptions of choto-san was the diagnosis of hypertension, with an odds ratio of 89.4 (95% confidence interval 28.85-277.11, p<0.001) (Table 5). These results of logistic regression analysis suggest that hypertension underlying headache, head discomfort, and dizziness were the main targets of choto-san.

**Table 3:** Good outcomes by medication Choto-san and antihypertensives are significantly more effective for clinical improvement than other treatments (logistic regression analysis). OR: odds ratio; AHT: antihypertensive; MM: migraine medicine; HM: headache medicine P<0.05 is denoted by an asterisk, and p<0.01 is denoted by a double asterisk.

	OR	95% confidence interval		p-value
Choto-san	3.13	1.83	5.35	p<0.001**
AHT	5.50	1.24	24.4	p=0.025*
MM	1.21	0.48	3.07	p=0.679
HM	1.13	0.62	2.04	p=0.698
Other	1.71	0.94	5.56	p=0.068

**Table 4:** Symptom improvement after treatment Headache, dizziness, and migraine are significantly improved. OR: odds ratio; H: headache; Dis: head discomfort; Diz: dizziness; two: two or more symptoms among headache, head discomfort, and dizziness; M: migraine. P<0.05 is denoted by an asterisk, and p<0.01 is denoted by a double asterisk.

	OR	95% confidence interval		p-value
H	1.96	1.20	3.19	p=0.007**
Dis	2.45	0.94	6.37	p=0.067
Diz	1.95	1.02	3.70	p=0.042*
Two	0.68	0.24	1.91	p=0.464
M	4.73	1.03	21.7	p=0.046*

**Table 5:** The most important factor related to choto-san prescription The main reason for choosing choto-san was hypertension, not each symptom complaint. OR: odds ratio; H: headache; Dis: head discomfort; Diz: dizziness; two: two or more symptoms among headache, head discomfort, and dizziness; M: migraine. P<0.05 is denoted by an asterisk, and p<0.01 is denoted by a double asterisk.

	OR	95% confidence interval		p-value
H	0.1168	0.0457	0.2989	p<0.001**
Dis	0.2802	0.0676	1.1621	p=0.0796
Diz	0.3476	0.1325	0.9122	p=0.0318*
Two	1.7702	0.3600	8.7048	p=0.4822
M	0.1179	0.0174	0.7973	p=0.0284*
Hypertension	89.4191	28.8541	277.1109	p<0.001**

## Discussion

Choto-san is made from choto-ko, sekko, bukuryo, hange, bofu, kikuka, ninjin, bakumon-to, chinpi, shokyo, and kanzo [1]. Choto-ko has a blood pressure-lowering effect [1] and acts on positive symptoms of the upper body (headache, hot flashes, dizziness, eye congestion, tinnitus, and shoulder stiffness, etc.) [2,3]. Ninjin and bakumon-to are thought to be suitable for elderly patients because of their moisturizing nature [2,3]. Kimura et al. reported that choto-san was effective for hypertension patients’ headaches and morning headaches [4] and concluded from the data of 51 patients that choto-san could be prescribed for headaches accompanying “morning headache," “sublingual vein distension," “cervical or shoulder stiffness," or “dizziness” and "unsteadiness" [5]. Gono demonstrated that choto-san was effective for “morning head dullness of hypertensive middle-aged or elderly patients” and “headache along with dizziness or tinnitus" [6]. According to Hirose et al., choto-san was effective against the following symptoms: headache, shoulder stiffness, dizziness, eye symptoms, hot flashes, sleeping disorder, tinnitus, and headache on waking up [7]. Kano et al. stressed the antihypertensive nature of choto-san [8], and the effectiveness of choto-san for “headache of hypertensive elderly patients” was reported by Mitsufuji et al. [9]. Saito reported that choto-san was effective for tinnitus in patients aged over 70 years [10]. The effectiveness of choto-san for eye pain after herpes zoster with hypertension, refractory hypertension after subarachnoid hemorrhage, and prolonged tinnitus was shown by Fukuda et al., Goto et al., and Nishida et al., respectively [11-13]. In each paper, the relationship between the symptoms assessed and hypertension was commonly discussed. Shiota et al. discussed the efficacy of choto-san for hypertension in their field of gynecology and stressed that its symptoms (dizzy head, shoulder stiffness, morning headache, and dizziness) were effectively treated by choto-san [14]. Tanaka et al. stated that choto-san blindly prescribed for refractory tinnitus was effective in more than 50% of patients. The authors speculated that the effectiveness of choto-san would be increased by compliant prescriptions according to Kampo diagnostics for choto-san [15].

Several authors demonstrated that choto-san-treated hypertensive rats had a longer lifespan than non-treated ones and also revealed its potential for preventing cerebrovascular disease [16-19]. The improvement of brain microcirculation and vascular endothelium and a nerve-protective effect were reported by some authors [20-22]. These favorable effects were ascribed to the action of choto-ko. Dohi et al. pointed out the no radical-scavenging activity of choto-san [23].

Based on these reports, choto-san is effective for various symptoms when hypertension might be the underlying cause behind these symptoms.

In the present study, the relationship between hypertension and choto-san was significant, and the outcome was good for patients who were prescribed choto-san. Choto-san may be effective for headaches, head discomfort, and dizziness with hypertension as the underlying basis and may contribute to the mitigation of subjective complaints. Therefore, it may be possible to conclude that choto-san was not prescribed for specific symptoms but for the existence of hypertension.

Headache, head discomfort, and dizziness are very common complaints of patients seen in neurosurgical outpatient departments. General headache medicines were prescribed in only 26.9%, and choto-san, migraine medicine, or other medication was prescribed in three-quarters of cases in the present study. The symptoms accompanying hypertension may respond to choto-san. Therefore, the correct diagnosis of the presence or absence of hypertension may contribute to patients’ good outcomes. A detailed interview eliciting the patients’ medical history and desirable medications for expected improvement may be related to the good outcomes of patients with these complaints.

Symptomatic treatment of these patients’ complaints is not always desirable, and identifying the cause behind the complaints is essential. It may be especially important to focus on hypertension, which could then result in effective treatment. Because a strong association between choto-san and hypertension was suggested in the current study, the remission of these symptoms may lead to the enhancement of the patients’ recognition of hypertension and connect them to hypertension treatment later. Choto-san, a Kampo medicine, may be easier to take than antihypertensives for patients complaining of these symptoms who do not think they have hypertension. Most Japanese patients recognize Kampo medicine as having milder effects than general antihypertensives and therefore not hesitate to take it as a first prescription for their complaints. In addition, the follow-up of such patients after prescription is very important, which may result in effective treatment.

Limitations

The current study has some limitations. First, it was retrospective and only provided limited evidence. Large-scale and prospective studies are needed to confirm our present data. Second, the results were obtained solely from patients attending our neurosurgical outpatient department, and the potential of hypertensive patients being included may be high. For example, migraine is mainly treated in the headache outpatient clinic, whereas cervical spondylosis may be predominant in the spinal cord outpatient division. Third, the number of candidates (171) was too small to detect the proof of choto-san effectiveness, and there is a lack of data to be compared with our data. Therefore, the distribution of prescriptions or patients in our institution may be limited.

## Conclusions

Hypertension was more common than expected in patients complaining of headache, head discomfort, or dizziness. Choto-san may be easier to take than general antihypertensives for Japanese patients because of its nature as a Kampo medicine. Most patients who come to our department with chief complaints of headache, head discomfort, or dizziness at their first visit do not recognize their potential hypertension. The remission of their symptoms after choto-san administration may contribute to their receiving hypertension treatment afterward, and this may be more important than symptom alleviation itself. Finding the chief cause behind patients’ complaints through the medical history or family history is essential, and ascertaining the outcomes relating to various symptoms after the start of medication is important for improving the quality of medical treatment. By extension, it is possible that this will prevent cerebrovascular diseases.

## References

[1] Sasaki S (2023). Evidence specifics: Chinese herbal medicine (prescription) (Article in Japanese). JOHNS.

[2] Raimura M (2021). When and how to use Chinese herbal medicine for migraines (Article in Japanese). Yakkyoku.

[3] Raimura M (2021). Headache × goshuyuto, chotosan, kakkonto, etc. (Article in Japanese). Yakkyoku.

[4] Kimura Y (2021). Chinese herbal medicine treatment for headaches (Article in Japanese). Nihon Zutsu Gakkai Shi.

[5] Kimura Y, Shimizu S, Tanaka A, Fujii A, Kinebuchi A, Inaki K, Sato H (2008). Headache for which chotosan is effective: study using multivariate analysis (Article in Japanese). Kampo Med.

[6] Gono Y (2020). Chinese herbal medicine treatment for headaches and dizziness (Article in Japanese). Pharmacia.

[7] Hirose S, Hosono K, Sakaguchi H, Hosono S (1977). Clinical use of chotosan (Article in Japanese). Kampo Med.

[8] Kano T, Ishii K, Kurobe Y, Akutagawa K, Ando G (1983). Tyoto-san in essential hypertension (clinical studies, report 1: effects on blood pressure, and on serum lipids and electrolytes) (Article in Japanese). Journal of Medical and Pharmaceutical Society.

[9] Mitsufuji T (2021). Headache treatment using an Oriental medicine approach (Article in Japanese). Pain Clinic.

[10] Saito A (1998). Treatment of tinnitus with chotosan using headache and high blood pressure as indicators (Article in Japanese). Jibi Rinsho.

[11] Fukuda S, Nambu T, Takahashi H, Kuroki K, Nishiyama H, Mitsuma T (2010). Two cases of eye symptoms after herpes zoster dramatically improved with chotosan (Article in Japanese). Kampo Med.

[12] Goto Y, Nakahara Y, Tanigawa S, Takeuchi H, Nanto M, Hashimoto N (2018). A case of subarachnoid hemorrhage in which chotosan was effective for treatment-resistant hypertension (Article in Japanese). Neurosurgery and Kampo Medicine.

[13] Nishida S, Sato H (2011). A case in which chotosan showed rapid antihypertensive effects during the course of tinnitus treatment (Article in Japanese). Kampo Med.

[14] Shiota A, Hata T (2019). The effect of Kampo medicine on middle aged women with hypertension (Article in Japanese). Recent Progress of Kampo Medicine in Obstetrics and Gynecology.

[15] Tanaka K, Tsuda M, Konishi K, Chen CK (1981). The efficacy of chotosan for intractable tinnitus (Article in Japanese). Jibi Rinsho.

[16] Shimada Y, Yang Q, Yokoyama K (2003). Choto-san prevents occurrence of stroke and prolongs life span in stroke-prone spontaneously hypertensive rats. Am J Chin Med.

[17] Sugimoto A, Goto K, Ishige A, Komatsu Y, Miyamoto KI (2000). Effect of choto-san, a Kampo medicine, on the cerebral blood flow autoregulation in spontaneously hypertensive rats. Jpn J Pharmacol.

[18] Yang Q, Goto H, Shimada Y, Kita T, Shibahara N, Terasawa K (2002). Effects of choto-san on hemorheological factors and vascular function in stroke-prone spontaneously hypertensive rats. Phytomedicine.

[19] Shimada Y, Goto H, Terasawa K (2006). Chotosan and cerebrovascular disorders: Clinical and experimental studies. Journal of Traditional Medicines.

[20] Shimada Y (2012). Effects of chotosan on cerebrovascular disorders. Journal of Traditional Medicines.

[21] Watanabe H, Zhao Q, Matsumoto K (2003). Pharmacological evidence for antidementia effect of choto-san (gouteng-san), a traditional Kampo medicine. Pharmacol Biochem Behav.

[22] Goto H, Yang Q, Kita T, Hikiami H, Shimada Y, Terasawa K (2001). Effects of choto-san on microcirculation, serum nitric oxide and lipid peroxides in patients with asymptomatic cerebral infarction. Am J Chin Med.

[23] Dohi K, Aruga T, Satoh K, Shioda S (2004). Choto-san (Kampo medicine) for the treatment of headache. Headache.

